# Advances in the treatment of uveitis in patients with spondyloarthritis - is it the time for biologic therapy?

**Published:** 2018

**Authors:** Traian-Costin Mitulescu, Marius Trandafir, Monica-Gabriela Dimăncescu, Radu-Constantin Ciuluvică, Viorela Popescu, Denisa Predețeanu, Sînziana Istrate, Liliana-Mary Voinea

**Affiliations:** * Department of Ophthalmology, University Emergency Hospital, Bucharest, Romania; ** Department of Rheumatology, “Sf. Maria” Clinical Hospital, Bucharest, Romania; *** Department of Anatomy and Embryology, “Carol Davila” University of Medicine and Pharmacy, Bucharest, Romania; **** Ophthalmology Department, “Carol Davila” University of Medicine and Pharmacy, Bucharest, Romania

**Keywords:** acute anterior uveitis, spondyloarthritis, anti-TNF drugs

## Abstract

Spondyloarthritis (SpA) is a heterogeneous group of diseases that includes ankylosing spondylitis (AS), psoriatic arthritis (PsA), reactive arthritis (ReA), inflammatory bowel disease-associated spondyloarthritis (IBD-SpA), and undifferentiated spondyloarthritis (unSpA). This group of diseases shares several clinical, imaging, and genetic features; the integration of these diseases in the group of SpA is needed for an early diagnosis and a prompt treatment. Uveitis is the most common extra-articular manifestation of SpA. HLA-B27-associated acute anterior uveitis (AAU) is the most frequent form of uveitis encountered in the SpA group. The general prevalence of HLA-B27-associated AAU in the group of SpA is about 30% and the general prevalence of SpA in patients with HLA-B27-associated AAU is over 50%. There are several differences in the clinical picture and evolution of HLA-B27-associated AAU in patients with SpA and knowing this is very important for the best therapeutic decision. Tumor necrosis factor α (TNFα) is a very important mediator not only in the pathogenic mechanisms of SpA, but also in the immune reactions that characterize HLA-B27-associated AAU in SpA. There is much evidence of the role of TNFα in SpA and HLA-B27-associated AAU, multiple studies showing efficacy of anti-TNFα drugs not only on rheumatic manifestations but also on ocular involvement. Conventional therapy of HLA-B27-associated AAU with local or systemic glucocorticoids and immunosuppressive drugs (sulfasalazine, methotrexate, azathioprine, etc.) in order to diminish the ocular inflammation is associated with many side effects, some of them being very severe and even life threatening. Therefore, new treatments, especially biologic therapy with anti-TNFα drugs, open a new opportunity for the treatment of these patients. It is very important to emphasize that antibody anti-TNFα agents (infliximab, adalimumab, golimumab) may be more efficient than soluble receptors of TNFα (etanercept) in decreasing the risk of HLA-B27-associated AAU in patients with SpA. The aim of this review made by a group of ophthalmologists and rheumatologists with recent and fruitful experience regarding the anti-TNF treatment of uveitis in patients with SpA is to make the community of ophthalmologists aware of this biologic therapy and that it is the right time to use it.

**Abbreviations:** AU = anterior uveitis; AAU = acute anterior uveitis; AS = ankylosing spondylitis; ASAS = Assessment of SpondyloArthritis Society; DBP = vitamin D binding protein; ESSG = European Spondyloarthropathy Study Group; HLA-B27 = human leukocyte antigen B27; IBD = inflammatory bowel disease; PsA = psoriatic arthritis; ReA = reactive arthritis; SpA = spondyloarthritis; TLRs = Toll-like receptors; TNFα = tumor necrosis factor α; unSpA = undifferentiated spondyloarthritis

## Introduction

The realization of modern medicine that medical specialties are interconnected overlapped or just tangential to one another has brought together the respective specialties in the field of research and clinical practice. Thus, the cooperation between ophthalmologists and rheumatologists seems to have solved the problem of the diagnosis and treatment of uveitis in patients with spondyloarthritis (SpA) [**1**].

The term SpA refers to a concept that represents a group of interrelated disorders comprising ankylosing spondylitis (AS), psoriatic arthritis (PsA), reactive arthritis (ReA), inflammatory bowel disease (IBD)-related SpA (IBD-SpA) and undifferentiated SpA (uSpA). Phenotypically, these diseases have several features in common including inflammatory back pain, peripheral arthritis, enthesitis, dactylitis and extra-articular features such as uveitis, psoriasis, and IBD. From an imaging point of view, this group of diseases presents bone edema in the early stages and radiographic sacroiliitis associated with spine ankylosis in the late stages of the diseases. Genetically, the diseases are associated with a major histocompatibility complex class 1 antigen represented by human leukocyte antigen-B27 (HLA-B27). Even if diseases included in the group of SpA have proper classification criteria, this approach is needed to make an early diagnosis for an early treatment [**2**,**3**]. 

Diagnosis of SpA is based on criteria presented by the European Spondyloarthropathy Study Group (ESSG) (**Table 1**), the positive diagnosis including one entry criterion and at least one additional criterion. This set of criteria is intended to be used for patients with SpA at the very early stage of the disease for a correct treatment even with anti-TNF drugs in order to decrease the impact of structural damage that characterizes this group of diseases [**4**].

**Table 1:** The ESSG criteria for SpA [**4**]

Entry criteria	Inflammatory back pain
	1. Onset of back discomfort before the age of 40 years
	2. Insidious onset
	3. Persistence for at least 3 months
	4. Associated with morning stiffness
	5. Improvement with exercise
Additional criteria	Asymmetrical synovitis predominantly of the lower limbs
	1.Positive family history
	2. Psoriasis
	3. Inflammatory bowel disease
	4. Urethritis, cervicitis or acute diarrhea with one month before arthritis
	5. Buttock pain alternating between buttocks
	6. Enthesopathy
	7. Plain film radiographic evidence of sacroiliitis
*Abbreviations:* ESSG - European Spondyloarthropathy Study Group; SpA - spondyloarthritis.	

Based on the ESSG criteria, the incidence rates of SpA range between 0.48 and 63 per 100 000 inhabitants, while the prevalence rates vary between 0.001 and 2.5% [**5**].

Although uveitis is the most common extra-articular manifestation in SpA, its prevalence, and characteristics are not well established in the clinical practice. In order to examine them, a systematic literature review and an analysis of the abstracts of rheumatology scientific meetings were made. Among the 957 articles (29 877 patients), 36 articles (1 989 patients) allowed a calculation of the prevalence and 36 articles (1 989 patients) described its characteristics. The prevalence of uveitis in SpA was 32.7% and it varied with the type of SpA being 33.2% for AS and 25.1% for PsA [**6**].

HLA-B27-associated acute anterior uveitis (AAU) is the most frequent form of uveitis encountered in patients with SpA. A retrospective study carried out on a group of 504 Chinese patients with HLA-B27-associated AAU showed that SpA was found in 387 (76.8%) patients, AS occurred in 214 (42.5%) patients, with a significantly higher prevalence in males than in females (p < 0.001), and uSpA occurred in 150 (29.8%) patients, with a significantly higher prevalence in females than in males (p < 0.001) [**7**].

Data from a recent overview showed that the estimated frequency of SpA in patients with AAU is around 50%, whereas AAU in SpA has been reported in at least 30% of the cases. AAU may precede the clinical features of SpA, may be present at diagnosis, or may complicate the SpA clinical course [**8**].

Uveitis is now considered so important for diagnosing SpA that it has been labeled together with psoriasis, colitis, HLA-B27 etc. as a SpA feature and introduced by the Assessment of SpondyloArthritis Society (ASAS) into the classification criteria for axial [**9**] and peripheral [**10**] SpA. Accumulating evidences from many studies with many patients confirmed the superior performance of these ASAS SpA criteria with a high-pooled sensitivity (73%) and specificity (88%) [**11**].

## Characteristics of uveitis in SpA

Uveitis means the inflammation of the uvea (iris, ciliary body, and choroid). In 2005, the Standardization of Uveitis Nomenclature (SUN) Working Group tried to classify uveitis based on the location of inflammation, the onset, and the course of the disease in order to standardize the approach to reporting clinical data in uveitis research [**12**]. 

There are several features of uveitis in patients with AS in whom HLA-B27-associated AAU is the most frequent form of uveitis. Symptoms of uveitis include pain, light sensitivity, blurring of vision, redness of eye and floaters. Severe complications may include high intraocular pressure, cataract, or glaucoma and even atrophy of the eyeball possibly leading to permanent loss of vision [**13**].

Patients with HLA-B27-associated AAU should be questioned by the ophthalmologist about inflammatory low back pain and also evaluated for other clinical features of SpA. Since a prolonged delay in diagnosis is common among SpA patients and the occurrence of AAU may be the reason for their interaction with the medical care, the occurrence of HLA-B27-associated AAU presents a unique opportunity for the identification of such undiagnosed SpA patients [**14**]. 

A retrospective study carried out on a group of 203 Chinese patients (184 male, 19 female) with AAU associated with AS showed unilateral involvement in 55.2% of the patients and bilateral involvement in 45.3% of the patients. However, the inflammation rarely affected both eyes simultaneously. Men were affected more frequently than women and the disease took a more painful course. All types of uveitis manifested acute inflammation and the disease lasted for about one month. Nearly half of patients experienced a recurrence of the disease [**15**].

The most important risk factors for uveitis in patients with AS, mentioned by Sun et al. on a group of 390 patients of whom 38 (9.7%) patients had experienced one or more episodes of uveitis, were peripheral arthritis, hip-joint involvement and high levels of circulating immune complex and anti-streptolysin 0 antibodies [**16**].

Another study showed that unlike patients with AS in whom uveitis had a sudden onset and was anterior, episodic, and unilateral, uveitis in patients with PsA had an insidious onset and was posterior, continuous and bilateral, very similar to that encountered in IBD (**Table 2**). Patients with uveitis and PsA with axial involvement were more likely to be male and HLA-B27 positive than PsA patients with uveitis and peripheral arthritis. Complications of uveitis were similar in AS and PsA [**17**].

**Table 2:** Summary of characteristics of uveitis in AS, ReA, IBD and PsA [**17**]

disease	age of uveitis onset	M/ F	HLA-B27	lifetime chance of uveitis	pattern of uveitis
AS/ ReA	33	2/1	89%	20% to 40%	sudden onset, anterior, unilateral, episodic
IBD	37	1/4.5	46%	3% to 11%	insidious onset, posterior, bilateral, continuous
PsA	39	2.2/1	67%	7%	insidious onset, posterior, bilateral, continuous
*Abbreviations:* AS - Ankylosing Spondylitis; IBD - Inflammatory Bowel Disease; PsA - Psoriatic Arthritis; ReA - Reactive Arthritis					

## Pathogenic mechanisms of uveitis in spondyloarthritis

The pathogenic mechanism of HLA-B27-associated AAU in patients with SpA is not entirely known, but there is evidence that shows that it is immune-mediated. Several factors are involved in the pathogenic mechanisms of AAU in patients with SpA: HLA-B27, Toll-like receptors (TLRs), vitamin D and TNFα.

Many studies emphasize that 50-60% of AAU patients have HLA-B27 [**18**]. The exact role of HLA-B27 in the development of AAU is not precisely known, but several hypotheses with animal models have been proposed. Many cases of uveitis follow gram-negative bacillary dysentery or *Chlamydia* infection. These gram-negative organisms include *Shigella, Salmonella, Klebsiella,* and *Yersinia* species. Similarities with the gram-negative cell wall lipopolysaccharides present in these microbes may explain their immunogenicity. Animal experiments with rodents that have been genetically altered to express human HLA-B27 molecules show that bacterial infection of the gut predisposes to arthritis and a ReA–like syndrome. Also, chronic intracellular *Chlamydia* joint or eye infection might stimulate, via the HLA-B27 molecule, a CD8 T-cell effector mechanism activated to kill the infections, which coincidentally also injures the eye [**19**].

TLRs of the innate immune system play a key role in protection from the microbial infection point of view and may play a role in the pathogenesis of uveitis. It has been shown that TLR4 expression, for example, has been expressed by uveal antigen presenting cells in the human iris and ciliary body. This provides a novel mechanism by which microbial triggers could initiate the development of AAU and explain the apparent high sensitivity of the uvea to lipopolysaccharide, the ligand for TLR4. Changes in the expression and function of TLR4 and TLR2 have been observed in patients with active AAU, further supporting the potential pathogenic role of these recognition receptors in the development of AAU. These TLRs could provide the missing molecular link between the observation of microbial triggers and the development of AAU and other immune-mediated inflammatory disorders [**20**].

Immunomodulatory actions of vitamin D and an association between vitamin D deficiency and many autoimmune diseases have been reported. Besides suppressing the adaptive immune response by enhancing the development of anti-inflammatory Th2-cells and inhibiting the development of Th1-cells, vitamin D also plays a significant role in the innate immune response. It has been shown that vitamin D supplementation in vivo can enhance the expression of cathelicidin, an antibacterial peptide, involved in decreasing the risk of autoimmune diseases such as uveitis [**21**].

There are several studies showing the relationship between low levels of vitamin D and the risk of HLA-B27-associated AAU in patients with SpA. A case control study conducted in 223 patients with AS and 239 ethnically matched controls who were genotyped for 8 single-nucleotide polymorphisms in the vitamin D binding protein (DBP) that transports vitamin D and its metabolites showed that no significant association was found between susceptibility to AS and DPB polymorphism. In a subgroup analysis of patients with AS, G alleles at rs4752 showed an increased risk of uveitis [**22**]. 

Seeking to emphasize the importance of vitamin D in inducing AAU in patients with AS, the authors of the present review carried out a study on a group of 34 patients with AS, of whom 11 patients with AS and AAU (AAA AS) and 23 patients with AS without AAU (wAAU AS) and 18 controls, trying to determine 25-hidroxivitamin D levels, IL-8, and serum amyloid A (SAA). The results of our study demonstrated that contrary to wAAU AS patients, significantly diminished levels of vitamin D and elevated levels of IL-8 characterized AAU AS patients; in addition, SAA level was significantly higher in AS patients compared to controls [**23**].

Produced especially by activated macrophages, TNFα is a very important pro-inflammatory cytokine with multiple biological functions [**24**] involved in inflammatory response that characterizes not only rheumatic disease but also ocular involvement:

─ activation of endothelial cells with increased expression of adhesion molecules and local increase of leukocyte trafficking;

─ increase of local leukocyte accumulation with induction or maintenance of HLA class II expression;

─ induction of neoangiogenesis involved in the local damage;

─ release of other pro-inflammatory cytokines (including IL-1, IL-6, IL-23, etc.);

─ release of chemokines (including MCP-1, IL-8, etc.) involved in leukocyte accumulation;

─ hepcidin production responsible for acute-phase response.

There are several studies showing the reason for a role of TNFα in the pathophysiology of SpA and in HLA-B27-associated AAU, which represents the most important extra-articular manifestation of this group of diseases. For clinical practice, it is very important to mention other studies showing high expression of TNFα not only in serum and in joints involved in these rheumatic diseases but also in aqueous humor in patients with HLA-B27-associated AAU.

Braun *et al.* showed that TNFα mRNA and proteins were present in inflamed sacroiliac joints of AS patients [**25**]. Lange *et al.* reported significantly increased TNFα plasma levels in AS patients with a positive correlation with disease activity [**26**]. Furthermore, the levels of TNFα were elevated in the synovial fluid [**27**] and skin lesions [**28**] in PsA patients and the TNFα level correlated also with disease severity [**29**].

Starting from a 1990 experimental study showing that the intravitreal injection of TNFα induced inflammation in the rabbit eye characterized by dilation of blood vessels in the iris, disruption of the blood-ocular barriers, migration of inflammatory cells into the anterior chamber, more recent studies have shown elevated levels of TNFα in serum and aqueous humor of patients with uveitis [**30**]. Recently, a prospective study carried on a group of 23 patients with uveitis, who were stratified in two categories according to the presence or absence of a systemic rheumatic disease and who were sub-classified into those who were HLA-B27 positive and HLA-B-27 negative, and a control group of 16 patients with uncomplicated cataracts, showed that in uveitis, patients serum and aqueous humor concentrations of TNFα were higher compared to the control group. Moreover, the concentration of TNFα in the aqueous humor of HLA-B27 positive patients was significantly higher than in those who were HLA-B27 negative [**31**].

## Biologic treatment of uveitis in SpA

The new advances in the pathogenesis of SpA and the established fact that TNFα is the most important pro-inflammatory cytokine in inducing articular and extra-articular manifestations of this group of diseases open new therapeutic perspectives not only for rheumatic diseases but also for ocular conditions involved. Romanian Guideline for Treatment of SpA patients with biological therapy established that patients with nonresponsive disease forms of AS and PsA to conventional therapy can benefit from biologic therapy not only with anti-TNFα antagonists but also with anti-IL17 drugs (**Table 3**) [**32**].

**Table 3:** Characteristics of biologic drugs approved for treatment of AS and PsA in Romania [**33**]

generic	trade	target	route	dosage	side effects
infliximab	Remicade® Remsima® Inflectra®	TNFα	iv	3-5 mg/ kg loading at weeks 0, 2, and 6, then maintenance 3-10 mg/ kg every 4-8 weeks (maximal dose 20 mg/ kg in children)	susceptibility to infections, including reactivation of tuberculosis, histoplasmosis, hepatitis B and fungal infection; hypersensitivity reactions; demyelinating disease; lupus-like syndrome; malignancy
adalimumab	Humira®	TNFα	sc	40 mg every 1-2 weeks (if weight < 30 kg: 20 mg every 2 weeks)	
etanercept	Enbrel® Benepali®	TNFα, TNFβ	sc	adults 50 mg weekly; children 0,8 mg/ kg/ week (maximum 50 mg/ week)	
golimumab	Simponi®	TNFα	sc	50 mg weekly	
certolizumab	Cimzia®	TNFα	sc	400 mg at weeks 0, 2 and 4 then 200 mg every 2 weeks or 400 mg every 4 weeks	
secukinumab	Cosentyx®	IL-17	sc	150 mg at weeks 0, 1, 2, 3 and 4, followed by monthly maintenance dosing	
*Abbreviations:* AS - Ankylosing Spondylitis; IL – interleukin; iv – intravenous; sc – subcutaneous; TNF – tumor necrosis factor					

In accordance with the 2016 update of the ASAS-EULAR management recommendations for axial SpA, “the rheumatologist is the coordinator in a multidisciplinary network because he has extensive knowledge of the entire disease spectrum of SpA” [**33**].

There are many old and new studies showing the efficacy of anti TNFα drugs on uveitis in patients with SpA or AS. 

The effect of TNF antagonists on AAU in patients with SpA or AS was analyzed in a large retrospective study and a meta-analysis of seven clinical trials, four of which were placebo-controlled, randomized trials. The retrospective study suggested that the TNF antagonists, infliximab and adalimumab, reduced the rate of AAU, whereas the frequency of AAU flares in patients with SpA who were treated with etanercept remained unchanged [**34**]. In the meta-analysis, infliximab and etanercept therapies reduced the incidence of AU flares and infliximab appeared to be more effective than etanercept; adalimumab was not evaluated [**35**].

Analyses of data from the “Review of safety and effectiveness with Adalimumab in patients with active ankylosing Spondylitis inhibitors” (RHAPSODY) trial carried out on a group of 1250 patients with ophthalmologist-diagnosed AU and active AS evaluated the effect of adalimumab 40 mg every other week for 20 weeks on AU flares. 

The study compared the rate of AU flares per 100 patient years (PYs) reported during the year before adalimumab treatment with rates during adalimumab treatment, in total and by patient subgroups. The AU flares rates before adalimumab were 15/ 100 PYs in all 1250 patients, 68.4/ 100 PYs in 274 patients with a history of AU flares, 176.9/ 100 PYs in 106 patients with a recent history of AU flares, 192.9/ 100 PYs in 28 patients with symptomatic AU at baseline and 129.1/ 100 PYs in 43 patients with a history of chronic uveitis. During adalimumab treatment, the rate of AU flares was reduced by 51% in all patients, by 58% in 274 patients with a history of AU, by 68% in 106 patients with a recent history of AU, by 50% in 28 patients with symptomatic AU at baseline and by 45% in 43 patients with chronic uveitis. 

Results of this prospective, open-label study suggested that adalimumab had a substantial preventive effect on AU flares in patients with active AU including patients with recently symptomatic AU and patients with chronic uveitis [**36**]. 

A study using real-world data from the Swedish Biologics Register compared the effect of adalimumab, etanercept and infliximab on AU occurrence in AS. For each TNFα inhibitor (TNFi) treatment, AU rates 2 years before TNFα start and for the first 2 years on TNFα treatment were compared. The study was carried out on a group of 1365 patients with AS and 406 patients were treated with adalimumab, 364 patients received etanercept and 605 patients were treated with infliximab. Compared with pretreatment rates, the study demonstrated a reduction in overall AU rates for adalimumab and infliximab and an increase for etanercept. The adjusted hazard ratio for AU in 1127 patients who were free for AU in the last 2 years before TNFi start were significantly higher for etanercept versus adalimumab and for etanercept versus infliximab. These data suggested differences in the effect on AU risk between adalimumab, etanercept and infliximab with a clear advantage for adalimumab and infliximab over etanercept [**37**].

Recently, a study carried out on 15 eyes of 12 HLA-B27 positive AS patients with resistant AU who received golimumab 50 mg per week showed that remission on AU was observed in 12 eyes of 15 and the visual acuity was significantly increased [**38**].

## Future perspectives

In Romania, the active and severe forms of AS and PsA, which did not previously respond to conventional therapies, are now treated with reimbursed biologic drugs according to the therapeutic protocols approved by the Ministry of Health and the National Health Insurance House. The patients belonging to this group of active and severe form of AS and PsA, also suffering from various degrees of uveitis, experienced improvement not only regarding joint involvement but also eye disease.

Among biologic drugs indicated in AS and PsA associated with uveitis, anti-TNF therapies (anti-TNFα antibodies, soluble receptors of TNFα) are the most important. Similar to those in literature, our clinical experience has demonstrated that anti-TNFα antibodies (infliximab, adalimumab, golimumab) are more effective than soluble receptors of TNFα (etanercept) in the treatment and prevention of uveitis flares in patients with AS and PsA.

More extensive studies on patients with HLA-B27-associated AAU not related to SpA are necessary, so that this category of patients may also benefit from biologic therapy with anti-TNFα drugs considering the reliable results obtained so far.

## Conclusions

1. AAU is the most common extra-articular manifestation in patients with SpA, its evolution depending of the duration of the disease and the presence of HLA-B27.

2. TNFα has a significant role in the pathogenic mechanism not only for AAU but also for SpA, both of which are targets for biologic therapy.

3. Efficiently used by rheumatologists, the anti-TNFα biologic treatment has become increasingly important for ophthalmologists in the treatment of uveitis in SpA patients.

4. Anti-TNFα antibodies (infliximab, adalimumab, golimumab) are preferential drugs versus soluble receptors of anti-TNFα (etanercept) in the treatment of AAU in patients with SpA.

5. The cooperation between ophthalmologists and rheumatologists is mandatory for a good management of patients with SpA and AAU.

**Conflict of interests**

The authors declare that there is no conflict of interests. All authors have equal contribution to this paper.
